# Role of Oxidant Scavengers in the Prevention of Ca^2+^ Homeostasis Disorders

**DOI:** 10.3390/molecules15107167

**Published:** 2010-10-15

**Authors:** Carmen Galan, Isaac Jardín, Natalia Dionisio, Ginés Salido, Juan A. Rosado

**Affiliations:** Cell Physiology Research Group, Department of Physiology, University of Extremadura, Caceres, 10071, Spain

**Keywords:** reactive oxygen species, scavengers, antioxidants, intracellular Ca^2+^ homeostasis

## Abstract

A number of disorders, such as Alzheimer disease and diabetes mellitus, have in common the alteration of the redox balance, resulting in an increase in reactive oxygen species (ROS) generation that might lead to the development of apoptosis and cell death. It has long been known that ROS can significantly alter Ca^2+^ mobilization, an intracellular signal that is involved in the regulation of a wide variety of cellular functions. Cells have a limited capability to counteract the effects of oxidative stress, but evidence has been provided supporting the beneficial effects of exogenous ROS scavengers. Here, we review the effects of oxidative stress on intracellular Ca^2+^ homeostasis and the role of antioxidants in the prevention and treatment of disorders associated to abnormal Ca^2+^ mobilization induced by ROS.

## 1. Calcium Homeostasis

In eukaryotic cells, Ca^2+^ is the most versatile signal involved in the control of cellular processes and functions [1,2,3]. This versatility derives from the fact that Ca^2+^ signalling works in a variety of ways, and the processes involved in Ca^2+^ mobilization are widely dynamic in range and amplitude. For example, in the cardiac myocyte, Ca^2+^ entering through L-type Ca^2+^ channels leads to a signal known as ‘spark’ that triggers contraction within microseconds; on the other hand, the duration of processes like gene transcription or cell proliferation ranges from minutes to hours. 

Cytosolic Ca^2+^ concentration ([Ca^2+^]_c_) is determined by a balance between the mechanisms that introduce Ca^2+^ into the cytoplasm, termed “on”, and those that remove it, termed “off”. These processes combine the action of a variety of channels, both in the plasma membrane and in the membrane of the intracellular stores, such as the endoplasmic or sarcoplasmic reticulum, including Ca^2+^ pumps, exchangers, and buffers. Many components of this large set of molecules have different isoforms with different characteristics and properties, which gives them the ability to make the system extremely versatile [4]. In the extracellular medium, the free Ca^2+^ concentration is about 1 mM, while in resting cells [Ca^2+^]_c_ is approximately 100 nM and in certain intracellular organelles, such as the endoplasmic reticulum (ER) or sarcoplasmic reticulum (SR), the free luminal Ca^2+^ concentration ([Ca^2+^]_L_) is around 0.2–1 mM; therefore, there is a clear concentration gradient between compartments that is essential for the regulation of the cellular processes in which Ca^2+^ participates [5]. In order to maintain these Ca^2+^ gradients, a strong homeostatic mechanism acts in the cell. 

Agonist-induced changes in [Ca^2+^]_c_ is determined by the balance between the Ca^2+^ “on” mechanisms, including Ca^2+^ influx from the extracellular medium and the output from intracellular stores, and Ca^2+^ “off” mechanisms, involving Ca^2+^ extrusion across the plasma membrane and sequestration into the stores or mitochondria [4]. The Ca^2+^ “off” mechanisms involve four different transporters: the plasma membrane Ca^2+^ ATPase (PMCA), that mediates Ca^2+^ extrusion across the plasma membrane, the sarco/endoplasmic reticulum Ca^2+^ ATPase (SERCA), which reintroduce Ca^2+^ into the ER/SR, the Na^+^/Ca^2+^ exchanger (NCX) that participates in cytosolic Ca^2+^ clearance through its exchange by Na^+^ and the mitochondrial Ca^2+^ uniporter (MCU), which that transports Ca^2+^ across the inner mitochondrial membrane. The two ATPases (PMCA and SERCA) carry out such transport using the energy provided by ATP hydrolysis. Four PMCA isoforms have been described in humans, all with similar molecular structure, consisting of ten membrane-spanning segments and five extracellular domains while their amino and carboxyl termini are located within the cell [6]. There are three different types of SERCA genes identified, giving rise to three SERCA isoforms [7]. These isoforms can be co-expressed in the same cell type, which could be related to the co-existence of different types of Ca^2+^ pools in the same cell [8]. Phosphorylation of an aspartic acid residue results in SERCA conformational changes that leads the enzyme to capture in a first step two Ca^2+^ per ATP molecule hydrolyzed, and then release them inside the ER/SR lumen [9]. In addition, when [Ca^2+^]_c_ reaches the micromolar range, both NCX and MCU can also transport Ca^2+^. NCX is a bidirectional transporter that combines the movement of three Na^+^ ions towards the cytosol with the transport of one Ca^2+^ in the opposite direction. NCX participates in the regulation of [Ca^2+^]_c_ in a number of cell types acting either in forward mode, as explained above, or in reverse mode, introducing Ca^2+^ in the cell when the Na^+^ concentration at the inner face of the plasma membrane substantially increases. Mitochondrial membrane potential confers the driving force necessary for the activity of the MCU. The threshold [Ca^2+^]_c_ for the activation of these four transporters is different as well as the transport rate. The lowest threshold is for the PMCA, which, by the way, has the lowest transport rate. This characteristic confers to PMCA an important role in maintaining resting [Ca^2+^]_c_ [10]. SERCA has lower affinity for Ca^2+^ but higher transport rates than PMCA [11]. On the other hand, the threshold [Ca^2+^]_c_ for NCX and MCU is the highest but they also display much higher transport rates than the ATPases. 

Agonist-activated Ca^2+^ “on” mechanisms includes Ca^2+^ release from the intracellular pools and entry through plasma membrane channels. There are three major types of intracellular Ca^2+^ channels responsible for Ca^2+^ release from the ER and the SR: the inositol 1,4,5-trisphosphate receptor (IP_3_R), the ryanodine receptor (RyR) and the nicotinic acid-adenine dinucleotide phosphate (NAADP) receptor. Mammalian cells possess three different isoforms for IP_3_R and RyR. Occupation of phospholipase C (PLC)-coupled membrane receptors by agonists results in the activation of phosphoinositide-specific PLC. As a result, inositol 1,4,5-trisphosphate (IP_3_) is generated and activates IP_3_R leading to Ca^2+^ efflux from the ER [12]. Ca^2+^ stored in the ER can also be released through RyR. The RyR is structurally and functionally similar to IP_3_R and is activated by cyclic ADP-ribose. These two channels are sensitive to Ca^2+^ itself, a phenomenon that underlies Ca^2+^-induced Ca^2+^ release and contributes to the rapid rise in [Ca^2+^]_c_ upon agonist stimulation and the development of regenerative Ca^2+^ waves. Finally, NAADP-mediated Ca^2+^ efflux from intracellular stores involves endolysosomal two-pore channels (TPC), especially TPC1 [13].

Agonist-induced Ca^2+^ entry from the extracellular medium occurs through a variety of Ca^2+^ channels in the plasma membrane depending on the cell type, which are gated by voltage (only in electrically excitable cells), agonists or second messengers [14,15]. In addition to these signals, other physical stimuli, such as mechanical stretch, are able to induce Ca^2+^ entry [16]. Special attention deserves a Ca^2+^ entry mechanism regulated by the filling state of the Ca^2+^ stores known as store-operated Ca^2+^ entry (SOCE) or capacitative Ca^2+^ entry. In 1986, Putney proposed that depletion of the intracellular Ca^2+^ stores lead to a sustained influx of Ca^2+^ through the plasma membrane independently of the elevation of [Ca^2+^]_c_ [17]. Subsequently, using biophysical techniques such as patch clamp, the existence of store-operated Ca^2+^ channels has been demonstrated, which can be opened in response to store depletion by various agents [18]. These channels, known as CRAC (Ca^2+^ release-activated Ca^2+^) channels, those mediating the highly Ca^2+^ selective current *I*_CRAC_, and SOC (store-operated channels), those conducting the non-selective cation current *I*_SOC_, have been characterized electrophysiologically [19,20,21,22]; however, their molecular identities have remained elusive for almost two decades. The Orai1 protein is the most relevant candidate to form the pore of the CRAC channels [23]. This protein has been demonstrated to form multimeric ion channel complexes in the plasma membrane [24]. The multimeric structure of the channel has recently been demonstrated as a tetramer. The pore structure consists of four separate units of Orai1, where charged residues are essential for Ca^2+^ selectivity [25]. Transient receptor potential proteins, alone or in combination with Orai1, have been reported to form the SOC channels, with lower Ca^2+^ selectivity than CRAC channels [26,27,28,29]. Especial attention has been given to the members of the canonical transient receptor potential (TRPC) subfamily, some of them can be activated by Ca^2+^ store depletion [30,31]. Orai1 and TRPC proteins can independently regulate ion current through CRAC channels and SOCs (*I*_CRAC_ and *I*_SOCs_) or might interact to form SOCs with different biophysical properties, thus providing the cell of some valuable tools to regulate specific Ca^2+^ signals (this has been extensively reviewed in [5]). SOC and CRAC channels have been reported to be sensitive to Ca^2+^ store depletion through the cooperative intraluminal Ca^2+^ sensor, STIM1 [32,33,34,35]. STIM1 is a transmembrane protein located in the Ca^2+^ stores that has been identified as the intraluminal Ca^2+^ sensor that communicates the amount of stored Ca^2+^ to plasma membrane channels [32,36]. This protein has a single transmembrane domain with an EF hand motif near the N-terminus, which is located in the lumen of the ER (or extracellularly when STIM1 is located in the plasma membrane [37]). A number of studies have demonstrated that the EF hand domain of STIM1, senses [Ca^2+^]_L_ and inhibits STIM1 activity when stores are filled. When a decrease in [Ca^2+^]_L_ occurs, Ca^2+^ dissociates from the EF hand motif and STIM1 activates Ca^2+^ channels [32,36,38,39].

## 2. Calcium Homeostasis Abnormalities Induced by Reactive Oxygen Species

ROS are small molecules that can be formed as a result of the normal aerobic metabolism [40], the activity of the immune system [41,42], the xenobiotic metabolism [43], or environmental pollution [44]. The sources of physiological ROS production are, among others, mitochondrial activity and the activity of enzymes such as xanthine oxidase, NADPH oxidase, cyclooxygenase and lipoxygenase. ROS were early classified as toxic; however, they have more recently been reported to act also as signalling molecules, in processes like transcription, gene expression or cell death [45,46,47]. At low concentrations ROS have been reported to act as secondary messengers in intracellular pathways involving Ca^2+^ mobilization, but at high concentrations they produce oxidative stress and cell damage [48,49,50,51].

The interrelationship between ROS production and Ca^2+^ homeostasis was first reported in the 70s. It is known that the involvement of the redox state in Ca^2+^ homeostasis is mediated by the modification of disulfide bonds between cysteine residues of some Ca^2+^ “off”-handling proteins, including PMCA, NCX and SERCA. Most studies reveal that ROS inactivates these transporters, leading to a rise in [Ca^2+^]_c_ and subsequent cell dysfunction. PMCA can be reversibly inactivated by ROS by an unclear mechanism that is suggested to be a reversible cysteine modification [52,53,54]. Current hypotheses propose that ROS alter the PMCA Tyr^589^, Met^622^ and Met^831^ residues [55]. Furthermore, there is evidence indicating that PMCA inactivation by ROS can be a protective system to avoid high consumption of ATP under stress conditions [56]. As a result of PMCA inactivation [Ca^2+^]_c_ rises over the resting value. 

ROS can also alter NCX function although the effect of ROS on NCX activity remains controversial. Hydrogen peroxide formed by the xanthine/xanthine oxidase system and superoxide anion increase the activity of NCX in myocytes [57]. However, in the same cells, oxidative stress induced by xanthine oxidase plus hypoxanthine inhibits the exchanger [58], and this exchanger has also been shown to be inhibited by the oxidant HOCl [59].

The effects of ROS on the activity of SERCA is also controversial. SERCA contains between 22 to 28 cysteine residues; therefore, the redox state is very important for its activity. ROS are capable of attenuating the activity of this pump *in vitro* by modifying sulphydryl groups [60,61,62]. Distinct SERCA isoforms show different susceptibility to ROS [51,60,61], which might be attributed to the different location of cysteine residues [63]. It has been hypothesized that small concentrations of ROS can stimulate this pump, whether high concentrations can inhibit SERCA [64], which has been reported to be more sensitive to ROS than PMCA [65]. 

Ca^2+^ “on” mechanisms are also susceptible to be altered by oxidative stress. Intracellular Ca^2+^ channels responsible for Ca^2+^ release from the intracellular stores are sensitive to ROS. IP_3_R function has been reported to be affected by ROS through the modification of cysteine residues. ROS increase the sensitivity of IP_3_R to cytosolic IP_3_ levels, thus IP_3_R might be sensitive to resting IP_3_ levels [66,67]. RyR can also been altered by changes in the redox state. The relationship between RyR channels and ROS production is probably the most widely investigated. In skeletal muscle, it has been suggested that the residue that confers ROS sensitivity to type 1 RyR (RyR1) is Cys^3635^. ROS play a dual role in RyR1 activity, being activated by concentrations of H_2_O_2_ between 100 μmol/L and 1 mmol/L [68] and inhibited by high concentrations of hydrogen peroxide (10 mmol/L) [69]. In cardiomyocytes, ROS produced by the activity of NOX enzymes increases type 2 RyR (RyR2) activity, interfering with its association with calmodulin (necessary to inhibit the channel) or FKBP12.6 (which stabilizes the channel) thus suggesting that binding of these two proteins to RyR2 channel is sensitive to the redox state [70]. Furthermore, studies in neurons have reported that ROS increase Ca^2+^ release mediated by type 3 RyR (RyR3) channels [70,71]. Modulation of RyR by ROS may be a mechanism of interaction between Ca^2+^ and the redox signalling pathways, and also a mechanism to increase or decrease Ca^2+^ signals as needed (for example, in neurons, ROS generation alters the activity of RyR channels, that causes long-term potentiation or depression, processes that depend on Ca^2+^ release through RyR) [54].

Finally, oxidants can also modulate the function of a number of Ca^2+^ permeable plasma membrane channels. ROS alter the activity of voltage-gated Ca^2+^ channels, specially the activity of L-type Ca^2+^ channels [72], which has been associated to the oxidation of SH groups resulting in altered Ca^2+^ entry in guinea pig ventricular myocytes [73]. A recent study has reported that exposure of cardiac myocites to hydrogen peroxide produces an increase of intracellular ROS and basal L-type channel activity [74]. Moreover, studies in human embryonic kidney 293 cells revealed that hydrogen peroxide increases basal L-type channel gating [75]. The effect of ROS on other voltage-gated Ca^2+^ channels has been less investigated. It has been shown that external application of hydrogen peroxide is able to activate voltage-dependent P/Q-type channels in neurons [76]. Evidence has also been provided in favour of an inhibitory role of ROS on voltage-dependent Ca^2+^ channel gating [73]. Although speculative, these discrepancies may be attributed to the different oxidants or the concentrations used.

In addition to voltage-gated Ca^2+^ channels, ROS can also affect the activity of other Ca^2+^ permeable channels, such as the channels conducting SOCE, receptor- or second messenger-operated Ca^2+^ entry. It has been reported that hydrogen peroxide decreases SOCE in thyroid cells through the activation of protein kinase C and not by a direct effect on SOCs and CRAC channels [77,78]. In human platelets, where ROS have been reported to play a physiological role in Ca^2+^ signalling, including SOCE [79], hydrogen peroxide plays a dual role in the activation of SOCE, with a stimulating effect at low concentrations (10-100 nM) and inhibitory effects at high concentrations (1 mM) [50]. Transient receptor potential channels have been shown to be sensitive to ROS [80]. Transient receptor potential canonical-3 (TRPC3)-forming channels are activated by ROS through the modulation of tyrosine phosphorylation [80] and transient receptor potential melastatin-2 (TRPM2), melastatin-7 (TRPM7) and ankyrin1 (TRPA1) channels are also sensitive to ROS. In neurons, TRPM7 and TRPM2, are activated by oxidative stress and participate in the pathophysiology of neurodegeneration [81,82]. In contrast, transient receptor potential polycystin-2 (TRPP2) channels are inhibited by oxidative stress in human syncytiotrophoblast [83]. The different effect of oxidative stress on TRP function might depend on the type of channel investigated and has been involved in the pathogenesis of a number of disorders. Other channels, such as Orai1, but not Orai3, have been shown to be inhibited by hydrogen peroxide-mediated oxidation. The differential redox sensitivity of these proteins has been attributed to the presence of an extracellularly located reactive cysteine, which is absent in Orai3 [84]. Oxidative stress also alters the ER Ca^2+^ sensor STIM1 and Ca^2+^ effectors such as Ca^2+^/calmodulin-dependent protein kinase II (for a review see [64]).

In summary, Ca^2+^ signalling is very sensitive to oxidants or reducing agents and changes in the redox state results in relevant changes in Ca^2+^ homeostasis, either altering Ca^2+^ mobilization from the internal stores and Ca^2+^ entry from the extracellular medium or modulating the activity of Ca^2+^ “off” mechanisms, including Ca^2+^ pumps and exchangers. The variable effects of ROS on Ca^2+^-handling mechanisms can be inhibitory or stimulatory, depending on the type of oxidant, its concentration and the time of exposure [85,86,87].

## 3. Disorders Caused by Reactive Oxygen Species and Therapeutic Strategies Based on the Use of Antioxidants

ROS have divergent effects on cellular function. At low concentrations, ROS have been reported to contribute to vascular tone regulation, mediate vasodilation, and regulate cell growth and differentiation, activation of platelet aggregation and stimulation of many kinases and proinflammatory genes [88,89,90,91,92]. On the other hand, oxidative stress promotes the development of a number of diseases, such as neurodegenerative, cardiovascular and metabolic diseases and certain types of cancer [90,93,94,95,96,97,98,99,100,101].

The controlled generation of ROS is necessary for many vital cellular functions. For instance, the response of macrophages to external agents leads to production of ROS and bioactive lipids derived from the metabolism of arachidonic acid [102,103,104]. A number of studies have shown that the imbalance between ROS generating and scavenging systems leads to oxidative stress which can cause oxidative damage to biomolecules, followed by various apoptotic pathways that lead to cell death [105,106,107]. In recent years, there has been an increasing use of antioxidants with the aim to regulate the redox balance [108,109] despite of only a few antioxidant are in use now in patients [110]. There are two disorders that illustrate the cellular dysfunction induced by abnormal Ca^2+^ homeostasis due to oxidative stress: diabetes mellitus and Alzheimer disease. 

Diabetes mellitus (DM) is a very common disease that affects over 180 million people, whose hallmarks are pancreatic β-cell dysfunction and insulin resistance [101]. Type 2 DM, which affects 90% of diabetics, leads to a number of cardiovascular alterations, including angiopathy, which is the main cause of morbidity and mortality in type 2 DM [111]. In the study of this disease, platelets have become a very important role because platelet hyperactivation and hiperaggregation play a key role in the development of angiopathy [112,113]. Platelets from diabetic patients have altered Ca^2+^ mobilization [114,115,116], increased ROS production [117,118] and enhanced protein tyrosine phosphorylation [119,120,121]. The reason of the enhanced ROS production during diabetes mellitus is not clear but it has been reported that diabetes mellitus is associated to hyperhomocysteinemia [122,123]. Studies in animal models have revealed that hyperhomocysteinaemia result in increased oxidative stress, impaired endothelial function and increased thrombogenicity. That rise in homocysteine concentration leads to increased production of ROS which can eventually trigger platelet hyperactivity [88,127] but lowering homocysteine levels by daily supplementation with antioxidants did not reduce the risk of developing type 2 DM [128].

Either at rest or after platelet stimulation with thrombin, [Ca^2+^]_c_ is higher in cells in patients with type 2 DM than in healthy donors [51,124], although platelets from healthy and type 2 DM subjects accumulate the same amount of Ca^2+^ into intracellular stores [119]. Abnormal Ca^2+^ homeostasis in platelets from type 2 diabetic patients has been attributed to altered Ca^2+^ extrusion mechanisms, increased IP_3_ generation or enhanced Ca^2+^ entry mechanisms [50]. Altered Ca^2+^ extrusion mechanisms have been reported for PMCA and SERCA, which are very sensitive to oxidative damage as mentioned above [51,115,125]. PMCA activity is regulated by tyrosine phosphorylation, and activation of platelets by thrombin stimulates Src-dependent PMCA tyrosine phosphorylation, and thus, inhibition of Ca^2+^ extrusion [119,126]. The increase in [Ca^2+^]_c_ can trigger the synthesis of thromboxane A2 and hyperaggregability causing platelet hyperactivation.There is evidence linking the decrease in vascular NO production with increased production of ROS that alter platelet function [127]. The generation of ROS in type 2 DM may result in platelet activation due to removal of the inhibitory effect of NO on platelet function. For example, superoxide anion and hydrogen peroxide are constantly produced in the cell, and diabetes is associated with a reduction in the production of antioxidants. High concentrations of ROS can alter platelet function by different pathways, including the activation of protein tyrosine phosphorylation by Bruton’s tyrosine kinases, and the Src family tyrosine kinases [79,128]. 

Different antioxidants has been shown to reverse DM-associated platelet hyperactivity and hyperaggregability by reducing [Ca^2+^]_c_ [117,129,130]. The use of antioxidants combined with diet, such as the Mediterranean diet or low-calorie diet and a healthy lifestyle provide big support in therapies for diabetes [131,132,133]. However, current studies are focused on the prevention of ROS production acting directly on ROS sources and possible treatments to reduce the deleterious effects of these oxidants through the development of inhibitors against the main sources of ROS. Mitochondria have been the focus of several studies aimed to treat or prevent cellular dysfunctions associated with oxidative stress [134,135]. For instance, the use of iron-chelators that attenuated hydrogen peroxide-induced mitochondrial membrane potential loss, decreased the release of cytochrome c into the cytoplasm and inhibited the activation of caspase-3, suggesting that these drugs may induce cytoprotective effects via the preservation of mitochondrial function [136]. The enzyme NADPH oxidase has been suggested as a possible target to decreasing ROS generation. A number of drugs used for the treatment of hypertension, hypercholesterolaemia and coronary artery disease such as the statins, AT1 (angiotensin II receptor type 1) antagonists and ACE inhibitors have been shown to decrease NADPH oxidase-derived superoxide and ROS production [137,138]. The use of NO donors suppressed vascular NADPH oxidase-dependent superoxide production have been probed successfully for oxidative stress attenuation [139,140].

### Neurodegenerative diseases

In healthy neurons Ca^2+^ is essential for neuronal development, synaptic transmission and plasticity, and the regulation of various metabolic pathways [141]. Glutamate, the major excitatory neurotransmitter in the central nervous system induced an increase in [Ca^2+^]_c_ directly by activating α-amino-3-hydroxy-5-methylisoxazole-4-propionate acid (AMPA) and N-methyl-D-aspartate (NMDA) receptor channels and indirectly by activating voltage-dependent Ca^2+^ channel. Besides, the activation of glutamate receptors coupled to the GTP-binding protein G_q11_ stimulates the release of IP_3_ leading to the opening of channels in the ER, following by a sustained entry of Ca^2+^ across the plasma membrane by store-operated or/and receptor-operated channels [142], and recent evidences suggest that transient receptor potential channels play an important role in neuronal Ca^2+^ homeostasis [143,144]. 

Under pathological conditions, the ability of neurons to control the increases in [Ca^2+^]_c_ is reduced, and this dysfunction can lead to neuronal death in three ways. First, direct or indirect cysteine proteases activation, such as calpain and caspases, which degrade a variety of substrates including cytoskeletal proteins, membrane receptors and metabolic enzymes. Calpain also has a role in the apoptotic cascade through its ability to activate caspases [145,146,147]. Second, Ca^2+^ induces oxidative stress through the activation of oxygenases in arachidonic acid metabolism, disturbance of mitochondrial Ca^2+^ and energy metabolism. ROS generated in response to Ca^2+^ influx induced by glutamate includes superoxide, hydrogen peroxide, hydroxyl radical and peroxynitrite [148]. Finally, Ca^2+^ induces apoptosis through activation of pro-apoptotic proteins such Bax, Par-4 and p53, enhancing mitochondrial membrane permeability and release of cytochrome *c* [149,150].

Oxidative stress plays a key role in the development of many neurodegenerative disorders such as Alzheimer’s and Parkinson’s disease. ROS induce blood-brain barrier disruption, mediate the transendothelial migration of monocytes and myelin phagocytosis, and finally induce cellular damage [151,152,153]. ROS also bind to lipids, proteins and nucleic acids leading to oxidative damage that ultimately leads to necrosis and cell death [154,155,156].

Alzheimer Disease (AD) is a disease that affects more than 35 million people worldwide. It is characterized by progressive loss of memory and other cognitive functions. Neurons progressively die and some areas of the brain show atrophy. The average life of AD patients is 8-10 years after its detection [157].

Alterations in Ca^2+^ homeostasis in neurons contribute to the neurodegenerative process. It have been shown that in AD patients the proteolytic processing of β-amyloid precursor protein and presenilin 1 and 2 are altered, leading to increased production of neurotoxic Aβ [158,159,160,161]. Amyloid β-peptide breaks the neuronal Ca^2+^ homeostasis by generating ROS, a process catalyzed by Cu^+^ and Fe^2+^. The neurotoxic Aβ induces oxidative stress, which leads to lipid peroxidation and breaks the Ca^2+^ homeostasis through the production of 4-hydroxy-2,3-nonenal, resulting in damage of the Na^+^/K^+^ and Ca^2+^-ATPases, and glucose and glutamate transporters [162]. Finally, the Aβ generated an increase in basal levels of intracellular Ca^2+^ that sensitizes neurons to apoptosis [163,164]. Besides breaking Ca^2+^ homeostasis by oxidative stress, Aβ oligomers can form pores for Ca^2+^ in cell membranes [165]. These findings suggest that treatments that regulate neuronal Ca^2+^ homeostasis might be able to prevent or at least delay the onset of AD. However, despite the success of drugs such as L-type Ca^2+^ channel blockers [166], therapies that disrupt Ca^2+^ fluxes may affect normal function of neurons, so the therapies should aim at stimulating the production of neurotrophic factors that protect neurons from cell death. Recent advances in AD treatment are focused on the use of the immune system. Current studies are mainly focused on the production of specific Aβ antibodies, on inhibitors of the enzymatic machinery involved in the production of Aβ from APP and also uncovered Aβ aggregation inhibitors [167,168,169].

Finally, because ROS can affect Ca^2+^ homeostasis, there are treatments that attempt to reduce oxidative stress in the cells, for instance inhibition of NADPH oxidase, responsible for most of the ROS production in microglia [170], which significantly reduces the development of the disease. The use of antioxidants, such vitamins A, C and E, or coenzyme Q, can form a protective barrier in the brain, preventing access of ROS and avoiding neuronal degeneration [171,172,173]. However, the use of other well known antioxidants, such as folic acid and vitamins B_6_ and B_12_ has been ineffective [174]. Currently, much attention has been focused on ROS generation by mitochondria, the main cellular source of ROS, with the aim to design different strategies that prevent the development of AD associated to stressful situations or aging [175,176].

Among other antioxidants, the use of flavonoids in therapeutic strategies has been extensively investigated. Flavonoids group a large and complex number of polyphenolic compounds that contain a three-ring structure with two aromatic centers and a central oxygenated heterocycle. On the basis of structural differences flavonoids are classified into flavonols, flavones, flavanones, catechins, anthocyanidins, isoflavones, dihydroflavonols, chalcones and proanthocyanidins [177]. The antioxidant actions of flavonoids has been reported to induce beneficial effects on a number of disorders. Flavonoids have been reported to reduce the risk of platelet-derived thrombotic disorders [178,179,180], an effect that has been attributed to the effect of flavonoids on a large number of signaling events, including prevention of lipid peroxidation [181], inhibition of actin filament polymerization [182], impairment of the thromboxane A2 signalling pathway [183,184] or reduction of Ca^2+^ mobilization [130,177,185,186]. Furthermore, flavonoids have been reported to exert vasodilatory effects, thus reducing coronary heart disease, through the inhibition of protein kinase C, cyclic nucleotide phosphodiesterases or Ca^2+^ uptake [187]. Plant flavonoids also exert modulatory effects on the immune response [188] and suppress pathways of lipid biosynthesis and of very low-density lipoprotein production, thus modulating lipid homeostasis [189]. Future studies of the biochemical mechanisms underlying the biological effects of flavonoids may reveal new strategies for the treatment of cardiovascular disease, as well as associated conditions such as obesity, hepatic steatosis, and Type 2 DM [189].

## 4. Conclusions

In summary, ROS have been reported to play an important functional role at physiological concentrations; however, when the concentration of oxidants exceeds the cellular scavenging mechanisms, ROS might be involved in the development of a number of cellular disorders, including abnormal Ca^2+^ homeostasis. The use of exogenous oxidant scavengers has been demonstrated to exert beneficial effects on a number of disorders, although further studies are necessary to design therapeutic strategies specific for the different diseases and scavengers. 
